# Predicting Distortion Magnitudes in Prussian Blue Analogues

**DOI:** 10.1021/jacs.3c08752

**Published:** 2023-11-06

**Authors:** John Cattermull, Mauro Pasta, Andrew L. Goodwin

**Affiliations:** † Inorganic Chemistry Laboratory, Department of Chemistry, University of Oxford, Oxford OX1 3QR, U.K.; ‡ Department of Materials, University of Oxford, Oxford OX1 3PH, U.K.

## Abstract

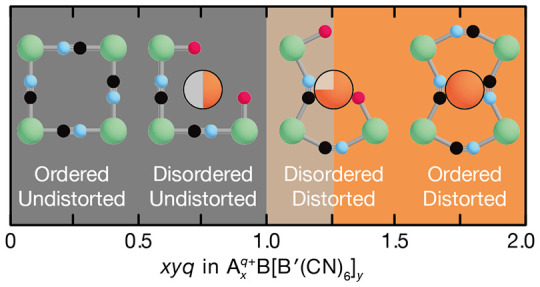

Based on simple electrostatic and harmonic potential considerations, we derive a straightforward expression linking the composition of a Prussian blue analogue (PBA) to its propensity to undergo collective structural distortions. We demonstrate the existence of a threshold value, below which PBAs are undistorted and above which PBAs distort by a degree that is controlled by a geometric tolerance factor. Our analysis rationalizes the presence, absence, and magnitude of distortions in a wide range of PBAs and distinguishes their structural chemistry from that of other hybrid perovskites.

Prussian blue analogues (PBAs) are an ever-topical family of materials with diverse functionality where structural distortions involving symmetry-breaking degrees of freedom play a key role in their physical and chemical properties.^1−4^ Local distortions mediate ion transport in the fast proton conductors M[Cr(CN)_6_]_2/3_·*z*H_2_O (M = Co, V), and magnetoelastic coupling (the interplay of magnetic order and lattice relaxation) governs a magnetic interference effect in the same systems.^1,5^ Correlated tilt distortions are key ingredient in the so-called “tilt-engineering” of novel ferroelectrics.^6−9^ And in the context of PBA battery materials, the *absence* of collective distortions during electrochemical cycling is considered important for the high reversible capacity of sodium manganese hexacyanomanganate electrodes.^10^ Hence, control over structural distortions is a crucial aspect of designing functional materials based on PBA chemistry.

In conventional perovskites, which share the same parent ABX_3_ network structure as PBAs, the existence and magnitude of structural distortions are usually rationalized in terms of the Goldschmidt tolerance factor^11,12^
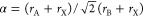
1This is a dimensionless geometric parameter that captures the extent to which the A-site cation fills the cavities of the anionic BX_3_ framework. A value of α = 1 implies perfect filling, which in turn prevents framework distortion. Decreasing the value of α generally induces volume-reducing distortions in the structure to compensate for the poorer geometric fit. Recognizing the relationship between tolerance factor and structural distortions has enabled targeted control over perovskite crystal structures, as in A_2_CrWO_6_ (A = Ba, Sr, and Ca ^13^), and is seen as a crucial design approach for optimizing functional response in, for example, magnetoresistive^14^ and photovoltaic^15^ perovskites.

Here we address the obvious and important question of whether an analogous geometric relationship holds for PBAs. While it is already well established that the tolerance factor approach might be successfully and straightforwardly extended to molecular perovskite analogues (“hybrid perovskites”),^16,17^ simple bonding considerations suggest the picture may be fundamentally different for PBAs in particular. The key distinction is that directional metal–cyanide interactions favor a linear linkage geometry,^18^ in contrast to the tilted geometry generally driven by covalency in conventional perovskites.^19^ For example, Mn[Pt(CN)_6_], which with no A-site cations at all has a tolerance factor well below 1, nonetheless adopts a cubic crystal structure in which the BX_3_ framework is undistorted.^20^ Instead it is by filling the A-site (i.e., increasing α) that distortions are switched on.^9,21^ This behavior contrasts with that of conventional perovskites but is conceptually similar to the activation of tilts in halide hybrid perovskites through hydrogen-bonding interactions between organic A-site cations and the anionic framework.^22^ An additional complication that we will address is that PBAs are highly nonstoichiometric frameworks, and one might expect partial site occupancies to play a nontrivial role in the activation or otherwise of collective distortions.

So why might filling the A-site of PBAs switch on structural distortions at all? The interaction between an A-site cation and its surrounding anionic BX_3_ framework is predominantly electrostatic, and since the corresponding attraction scales inversely with distance, it is intuitive that distortions reducing the A···X distance should be energetically favored (Figure 1a). However, it is not possible to reduce one A···X distance without increasing another. Consequently, the electrostatic driving force for distortion comes not from a direct reduction in the A···X separation *r*_AX_ by some distance *d*, say, but from the combined effect of electrostatic terms that vary alternately as 1/(*r*_AX_ + *d*) and 1/(*r*_AX_ – *d*). For small distortions *d* ≪ *r* = *r*_AX_, we have an electrostatic stabilization
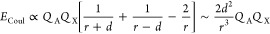
2that scales as the square of the distortion magnitude *d*^2^ (here *Q*_A_ and *Q*_X_ are the effective charges on the A- and X-site; see Supporting Information for further discussion). Hence for a PBA with formula A_*x*_^*q*+^B[B′(CN)_6_]_*y*_ we can write
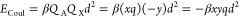
3where *x* and *y* are the A-site and X-site occupancies, *q* is the charge on the A-site cation, and β > 0 is a proportionality constant into which we have subsumed the 2/*r*^3^ factor of eq 2.^a^

**Figure 1:**
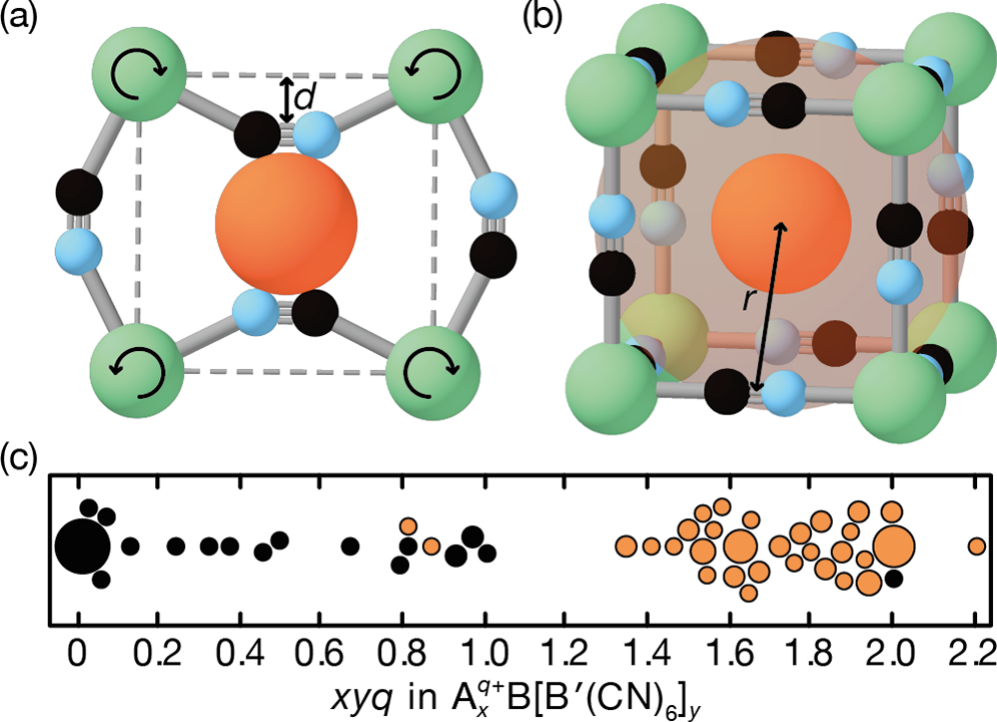
(a) Schematic representation of a generalized distortion in the PBA structure. The A-site cation (orange sphere) is attracted electrostatically to the 12 surrounding CN^–^ ligands (C in black, N in blue). Structural distortions, such as correlated tilts of the B-centered octahedra (B-site shown in green), reduce the A–X separation by the distance *d*. (b) The degree of mismatch between available A-site volume (translucent sphere) and actual volume occupied by the A-site cation (orange sphere) determines the maximum value of *d* accessible under deformation; the total distance between the centers of the A- and X-sites is given by *r*. (c) Scatter plot of *xyq* values for many different PBA samples taken from the literature, with symbols colored according to whether the corresponding structure is distorted (orange) or undistorted (black). Symbol size denotes the number of equivalent structures with a common *xyq* value.

Acting against this electrostatic driving force is the energy cost of distorting M–CN–M linkages from their linear ground-state. The leading (harmonic) term in the corresponding energy expansion has the form  Here *k* is an effective spring constant that reflects the stiffness of the M–CN–M linkages to deformation. So this energy cost also scales as *d*^2^ for small distortion magnitudes. Combining these two terms, one expects distortions to occur whenever *E*_Coul_ + *E*_harm_ < 0; that is,
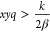
4Note that the dependence on *d* has now disappeared because both terms scale as *d*^2^.

We show in Figure 1c the distribution of *xyq* values for a large range of PBAs of different compositions (details in the Supporting Information). With only a very small number of exceptions (which we will come to discuss), we observe a partitioning into undistorted structures at low values of *xyq* and distorted structures at high values of *xyq* (using as our definition of “distorted” any departure from ideal cubic crystal symmetry). The existence of a universal critical value of *k*/(2β) for which eq 4 holds suggests that the effective stiffnesses *k* and Coulomb prefactors β (which depend on the A···X distance) do not vary too much among PBAs. From our data set this critical value is about 1.

This result provides a simple but powerful heuristic for estimating whether or not a given PBA of arbitrary composition A_*x*_^*q*+^B[B′(CN)_6_]_*y*_ is likely to be structurally distorted, based simply on the corresponding product *xyq*. Immediately it rationalizes why low-vacancy PBA cathode materials switch between distorted and undistorted phases as A-site cations are shuttled in and out during electrochemical cycling, for example.^23^ The reduced A-site occupancy of the oxidized state means there is an insufficient electrostatic driving force to drive distortion, but on reduction the increased A-site occupancy induces long-range symmetry breaking.^24^

Our derivation is intentionally simplistic and involves a number of crude approximations. We have not taken into account electronic distortion mechanisms, such as the Jahn–Teller effect, which needs to be treated separately. Indeed the two outliers at *xyq* ≃ 0.8 correspond to low-vacancy PBAs Rb_0.90_Cu[Co(CN)_6_]_0.92_ and Rb_0.85_Cu[Fe(CN)_6_]_0.95_ containing Jahn–Teller-active Cu^2+^. In the particular case of Rb-containing PBAs, which tend to show columnar Rb order at half-filling,^25^ the combination of A-site order and cooperative Jahn–Teller order from the Cu^2+^ ions automatically activates an additional tilt instability through symmetry considerations alone.^26^ Consequently, the presence of distortions in these two systems arises from electronic effects rather than from an electrostatic drive to reduce the volume. This point is made clear also in the small volume strains involved (<4%). We have also made the assumption that alkali cations are situated on (or near) the 12-coordinate A-site, which is almost universally true^18,27−29^ but is not necessarily the case for some Na-containing PBAs,^30^ nor is it for the related family of cyanoelpasolites.^31^ Mindful of these various caveats, we nonetheless see the value of understanding which general factors switch on and off collective distortions in different PBAs.

But what determines distortion magnitude? An implication of the analysis leading to eq 4 is that a system with *xyq* > 1 will minimize its energy by maximizing the value of *d*; in other words, it should distort as much as the structure allows (Figure 1b). This aspect is more obviously connected to the spirit of tolerance factor analysis, and we sought to establish a relationship between distortion magnitude and the value of α. We used the modified tolerance factor expression^32^
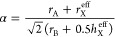
5in our analysis. Here, *r*_X_^eff^ is the effective radius and *h*_X_^eff^ the effective height of a rigid cylinder used to approximate the cyanide linker. We used values of *h*_X_^eff^ = 3.65 Å and *r*_X_^eff^ = 1.68 Å as previously applied for hybrid perovskites.^32^ Note that, in the case of PBAs, hydration might influence the appropriate value of *r*_A_, e.g., for [Na–OH_2_]^+^ species.^33,34^ Given the diversity of possible distortion mechanisms in PBAs,^2,35^ we take the magnitude of volume collapse as a generic, universal measure of distortion extent. To do so, we estimate an expected undistorted unit-cell volume *V*_ref_ on the basis of tabulated ionic radii^36^ and then calculate the relative distortion δ = (*V*_exp_ – *V*_ref_)/*V*_ref_.

We show in Figure 2 the relationship between α and δ observed for the various distorted PBAs in our data set. A clear linear trend emerges that links the extent of structural distortion to the departure of the modified tolerance factor from its ideal value of one. In this sense, the behavior of distorted PBAs is similar to that of conventional perovskites.^12^ Our data suggest an empirical relation δ ≃ 2(1 – α); e.g., a tolerance factor of 0.9 corresponds to a volume reduction of about 20%.

**Figure 2:**
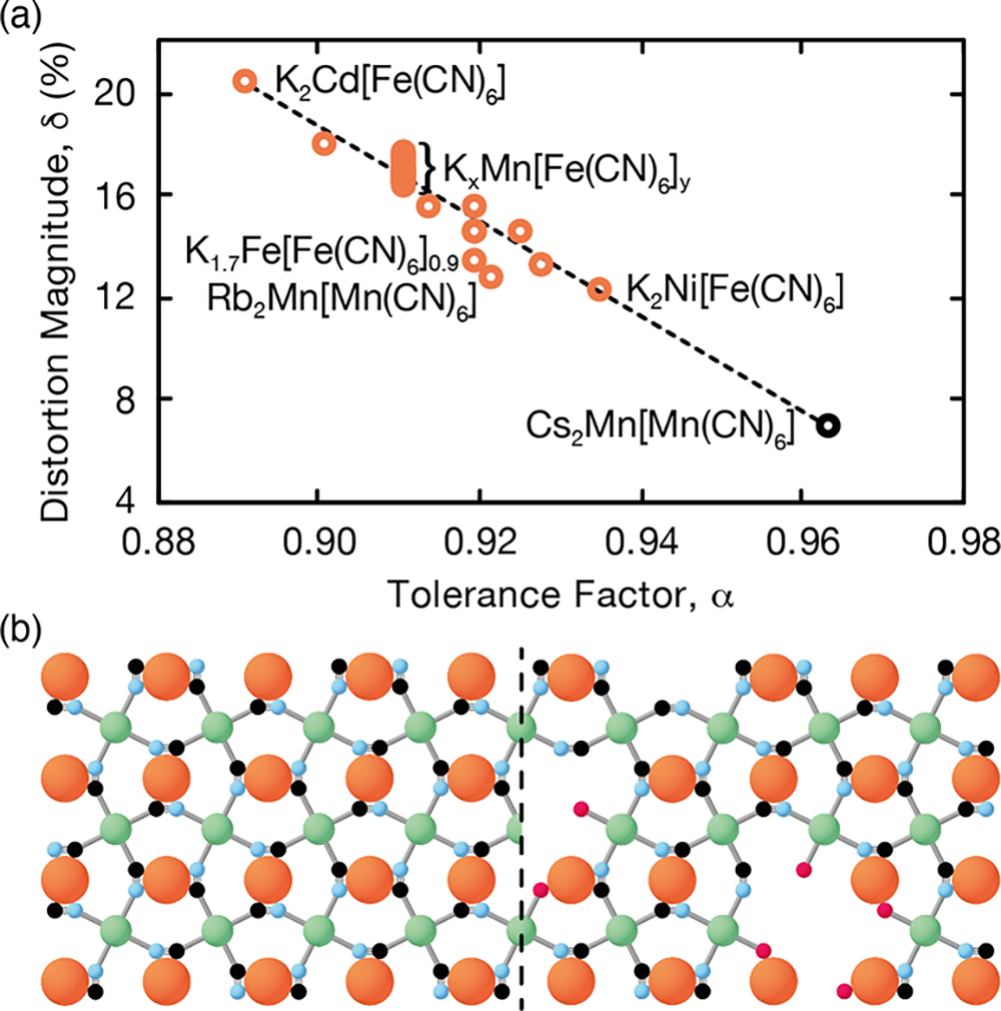
(a) Relationship between distortion magnitude and tolerance factor for those PBAs represented in Figure 1c with *xyq* > 1. (b) Low concentrations of vacancies on either A-site or B-site (right) have little effect on the magnitude of cooperative distortion, which is limited by the same geometric considerations at play in the parent, vacancy-free, structure (left).

We make a number of comments regarding Figure 2. Considering first the compositionally very different pair of materials Rb_2_Mn[Mn(CN)_6_] and K_1.7_Fe[Fe(CN)_6_]_0.9_, their similar distortion magnitudes can now be rationalized in terms of their similar tolerance factors (0.906 and 0.904, respectively). Second, because volume reduction is cooperative, we expect that the magnitude of distortion should be largely unaffected by the incorporation of A- or X-site vacancies (Figure 2b). It is known, for example, that vacancies do not affect the tilt instabilities of PBAs.^37^ Indeed, we find an entire family of 40 K_*x*_Mn[Fe(CN)_6_]_*y*_ systems (with many different vacancy fractions but identical tolerance factors) show remarkably little variation in distortion magnitude. The small variation in cell volume is clearly evidenced by the 24 K_*x*_Mn[Fe(CN)_6_]_*y*_ samples in ref (38). By contrast, replacing Mn by Ni or Cd (which changes α) results in a much more dramatic variation in δ. As a third point, we consider the system Cs_2_Mn[Mn(CN)_6_] originally flagged as the undistorted anomaly in Figure 1c. Here, *xyq* = 2, so one expects a distorted structure, but the tolerance factor is so near unity that the corresponding value of δ is only about 6%. We anticipate that the system is cubic because small distortion magnitudes can be accommodated in the flexible PBA structure through dynamic and/or short-range fluctuations rather than collective symmetry breaking.

The effect of external pressure is to include an additional *p*Δ*V* term to the lattice enthalpy balance, which behaves as if to reduce the effective spring constant *k* and so shifts the critical value of *xyq* to below 1. This effect is consistent with the experimental observation of pressure-driven structural distortions in some PBAs.^8,39^ The effect of temperature is formally given by the balance of entropic terms in the free energies, but one simplification is to consider the effective radii of eq 5 to be temperature-dependent. Since one expects the thermal volume of A- and X-site ions to grow more quickly than that of the B-site, α should increase with temperature, rationalizing the thermal quenching of distortions observed experimentally.^21,24^

So the structural chemistry of PBAs differs conceptually from that of other hybrid perovskites as a consequence of the linear ground-state geometry of the B–X–B linkages. In conventional perovskites, for example, the tilt modes are usually mechanically unstable in the absence of sufficiently large A-site cations.^40,41^ Hence *k* < 0 and the inequality of eq 4 always holds. The cyanide ion is relatively unusual in stabilizing linear connectivity since most other molecular linkers exploited in hybrid perovskites (e.g., azide,^42^ formate,^43^ or thiocyanate^44^) all favor distorted geometries. Hence the existence of compositional distortion thresholds is not universal but may nonetheless be relevant to dicyanometallates,^45^ borohydrides,^46^ bifluorides,^47^ and even metal–organic frameworks.^48,49^

From the perspective of functional PBA design, our results suggest a number of avenues. In cases where structural distortions are favorable (e.g., when targeting hybrid-improper ferroelectrics^8^) then the combination of high A-site occupancies, low hexacyanometallate vacancy fractions, and a tolerance factor much reduced from unity will collectively maximize the degree of distortion. Inverting these criteria then forms the design strategy for avoiding structural distortions, which may be important for improving the reversible capacity of PBA-based cathodes.

## Supporting Information Available

Details of samples used for Figure 1c and Figure 2a, and discussion regarding *E*_Coul_ (PDF)

## Footnotes

a The PBA symmetry is such that distortions of *d* and −*d* must give rise to the same variation in *E*_Coul_, and hence the leading term in any expansion of this energy will always be quadratic, irrespective of details such as the charge distribution along M–CN–M linkages or which particular tilt modes are activated.
