# Supplementary material for: Predicting Distortion Magnitudes in Prussian Blue Analogues
Source: J Am Chem Soc. 2023 Nov 6;145(45):24471–5. doi: 10.1021/jacs.3c08752 (PMC10655185; doi:10.1021/jacs.3c08752)
Supplement: Supplementary file 1 — ja3c08752_si_001.pdf [file ja3c08752_si_001.pdf]

Predicting Distortion Magnitudes in Prussian Blue Analogues

SUPPLEMENTARY INFORMATION

John Cattermull,[a,b] Mauro Pasta[b] and and Andrew L. Goodwin[a*]

[a]Department of Chemistry, University of Oxford, Inorganic Chemistry Laboratory, Oxford, OX1 3QR, U.K.

[b]Department of Materials, University of Oxford, Oxford, OX1 3PH, U.K.

*To whom correspondence should be addressed;

E-mail: andrew.goodwin@chem.ox.ac.uk

Contents

1 Details of samples **3**

2 Discussion regarding $\mathbf{E}_{\mathrm{Coul}}$ **6**

3 References **8**

1 Details of samples

Sample	xyq	V_{ref}	V_{exp}	δ (%)	α	Ref.
$K_{2.13}Mn[Fe]_{1.03}$	2.20	154.9	128.9	16.783	0.9112	S1
$K_{2.00}Cd[Fe]_{1.00}$	2.00	165.5	132.0	20.237	0.8913	S2
$K_{2.00}Mn[Mn]_{1.00}$	2.00	160.1	131.6	17.794	0.9011	S3
$K_{2.00}Mn[Fe]_{1.00}$	2.00	154.9	129.2	16.587	0.9112	S1
$K_{2.00}Fe[Fe]_{1.00}$	2.00	150.6	127.4	15.384	0.9198	S2
$K_{2.00}Co[Fe]_{1.00}$	2.00	147.6	126.4	14.395	0.9259	S2
$Rb_{2.00}Mn[Mn]_{1.00}$	2.00	160.1	139.8	12.662	0.9220	S3
$K_{2.00}Ni[Fe]_{1.00}$	2.00	143.1	125.7	12.101	0.9356	S2
$K_{1.99}Mn[Fe]_{0.94}$	1.99	154.9	129.1	16.618	0.9112	S1
$K_{1.99}Mn[Fe]_{1.00}$	1.99	154.9	129.5	16.383	0.9112	S1
$K_{1.96}Mn[Fe]_{0.99}$	1.94	154.9	128.7	16.897	0.9112	S2
$K_{1.96}Cu[Fe]_{0.99}$	1.94	146.4	127.2	13.081	0.9285	S4
$K_{1.94}Mn[Fe]_{1.01}$	1.91	154.9	127.7	17.549	0.9112	S1
$K_{1.94}Mn[Fe]_{0.994}$	1.91	154.9	128.3	17.147	0.9112	S1
$K_{1.93}Mn[Fe]_{0.98}$	1.90	154.9	129.3	16.493	0.9112	S1
$K_{1.92}Mn[Fe]_{0.98}$	1.88	154.9	129.1	16.650	0.9112	S1
$K_{1.88}Mn[Fe]_{0.97}$	1.82	154.9	129.5	16.393	0.9112	S1
$K_{1.87}Mn[Fe]_{0.97}$	1.81	154.9	129.2	16.582	0.9112	S1
$K_{1.86}Mn[Fe]_{0.96}$	1.79	154.9	128.9	16.729	0.9112	S1
$K_{1.84}Mn[Fe]_{0.99}$	1.77	154.9	128.1	17.286	0.9112	S1
$K_{1.84}Mn[Fe]_{0.96}$	1.77	154.9	129.0	16.706	0.9112	S1
$K_{1.83}Mn[Fe]_{0.96}$	1.75	154.9	129.0	16.687	0.9112	S1
$K_{1.80}Mn[Fe]_{0.95}$	1.71	154.9	129.0	16.692	0.9112	S1
$K_{1.80}Mn[Fe]_{0.95}$	1.71	154.9	129.5	16.372	0.9112	S1
$K_{1.77}Mn[Fe]_{0.94}$	1.67	154.9	129.1	16.627	0.9112	S1
$K_{1.76}Mn[Fe]_{0.94}$	1.65	154.9	128.8	16.838	0.9112	S1
$K_{1.75}Mn[Fe]_{0.93}$	1.64	154.9	129.2	16.586	0.9112	S1
$K_{1.74}Mn[Fe]_{0.94}$	1.63	154.9	128.6	16.959	0.9112	S1

$K_{1.74}Mn[Fe]_{0.94}$	1.63	154.9	128.7	16.898	0.9112	S1
$K_{1.74}Mn[Fe]_{0.94}$	1.63	154.9	128.7	16.858	0.9112	S1
$K_{1.74}Mn[Fe]_{0.94}$	1.63	154.9	129.9	16.126	0.9112	S1
$K_{1.72}Mn[Fe]_{0.93}$	1.60	154.9	129.0	16.720	0.9112	S1
$K_{1.72}Mn[Fe]_{0.92}$	1.60	154.9	129.5	16.353	0.9112	S1
$K_{1.69}Mn[Fe]_{0.92}$	1.56	154.9	129.5	16.392	0.9112	S1
$K_{1.68}Mn[Fe]_{0.92}$	1.55	154.9	128.9	16.734	0.9112	S1
$K_{1.67}Mn[Fe]_{0.92}$	1.53	154.9	128.8	16.822	0.9112	S1
$K_{1.64}Mn[Fe]_{0.91}$	1.49	154.9	128.6	16.926	0.9112	S1
$K_{1.64}Fe[Fe]_{0.89}$	1.49	150.6	129.0	14.321	0.9198	S1
$K_{1.58}Mn[Fe]_{0.85}$	1.41	154.9	129.0	16.702	0.9112	S1
$K_{1.52}Mn[Fe]_{0.88}$	1.34	154.9	128.9	16.785	0.9112	S1
$Cs_{2.00}Mn[Mn]_{1.00}$	2.00	160.1	149.1	6.854	0.9636	S3
$K_{1.94}Mn[Fe]_{1.0}$	1.94	154.9	127.7	17.552	0.9112	S5
$K_{1.84}Mn[Fe]_{0.99}$	1.82	154.9	128.1	17.284	0.9112	S5
$K_{1.99}Mn[Fe]_{0.94}$	1.87	154.9	129.1	16.618	0.9112	S6
$K_{1.94}Mn[Fe]_{0.99}$	1.92	154.9	128.3	17.147	0.9112	S7
$K_{1.75}Mn[Fe]_{0.93}$	1.63	154.9	129.2	16.586	0.9112	S8
$K_{1.72}Mn[Fe]_{0.92}$	1.58	154.9	129.5	16.353	0.9112	S7
$K_{1.58}Mn[Fe]_{0.85}$	1.34	154.9	129.0	16.702	0.9112	S5
$K_{1.85}Mn_{0.33}Fe_{0.67}[Fe]_{0.98}$	1.81	153.4	129.8	15.412	0.9140	S6
$K_{1.64}Fe[Fe]_{0.89}$	1.46	150.6	129.0	14.321	0.9198	S8
$K_{1.93}Fe[Fe]_{0.97}$	1.87	150.6	128.9	14.358	0.9198	S9
$K_{1.70}Fe[Fe]_{0.90}$	1.53	150.6	130.5	13.312	0.9198	S10
$K_{1.60}Mn[Fe]_{0.96}$	1.54	154.9	128.0	17.316	0.9112	S11
$K_{1.70}Mn[Fe]_{0.90}$	1.53	154.9	129.2	16.575	0.9112	S12
$Cs_{1.00}Mn[Co]_{1.00}$	1.00	149.3	144.8	2.995	0.9864	S13
$Cs_{0.97}Cu[Fe]_{0.99}$	0.96	141.4	141.2	0.178	1.0043	S14
$Rb_{0.94}Mn[Fe]_{0.98}$	0.92	149.7	147.2	1.657	0.9428	S15
$Rb_{0.90}Cu[Co]_{0.92}$	0.83	141.4	136.0	3.799	0.9609	S16
$Rb_{0.87}Mn[Co]_{0.91}$	0.79	149.3	143.6	3.824	0.9437	S13

$Rb_{0.85}Mn[Fe]_{0.95}$	0.81	149.7	146.9	1.909	0.9428	S15
$Rb_{0.85}Cu[Fe]_{0.95}$	0.81	141.4	139.7	1.248	0.9609	S14
$Rb_{0.73}Mn[Fe]_{0.91}$	0.66	149.7	146.1	2.410	0.9428	S15
$Co[Pt]_{1.00}$	0.00	148.9	147.3	1.046	0.4483	S17
$Ni[Pt]_{1.00}$	0.00	144.3	143.1	0.804	0.4530	S17
$Fe[Pt]_{1.00}$	0.00	151.8	151.1	0.491	0.4453	S17
$Cd[Pt]_{1.00}$	0.00	166.8	166.6	0.150	0.4316	S17
$Mn[Pt]_{1.00}$	0.00	156.2	158.3	-1.385	0.4412	S17
$Zn[Pt]_{1.00}$	0.00	148.5	150.8	-1.609	0.4487	S17
$Fe[Fe]_{0.75}$	0.00	128.4	144.5	-12.534	0.4709	S18
$Cu[Co]_{0.67}$	0.00	141.0	127.4	9.672	0.4565	S19
$Mn[Co]_{0.67}$	0.00	149.3	141.8	5.056	0.4479	S19
$K_{0.10}Mn[Co]_{0.70}$	0.07	149.3	142.1	4.812	0.9224	S20
$K_{0.40}Mn[Co]_{0.80}$	0.32	149.3	142.5	4.566	0.9224	S20
$Cu[Fe]_{0.70}$	0.00	141.4	130.0	8.081	0.4560	S21
$K_{0.67}Cu[Fe]_{0.68}$	0.46	146.4	126.8	13.342	0.9285	S21
$Rb_{0.98}Mn[Fe]_{0.94}$	0.92	149.7	147.3	1.588	0.9428	S22
$K_{0.03}Co[Fe]_{0.68}$	0.02	142.6	137.8	3.406	0.9365	S23
$Cs_{0.08}Co[Fe]_{0.7}$	0.06	142.3	138.6	2.609	1.0023	S23
$Cs_{0.18}Co[Fe]_{0.73}$	0.13	141.3	139.8	1.045	1.0047	S23
$Cs_{0.30}Co[Fe]_{0.80}$	0.24	137.8	131.9	4.282	1.0131	S23
$Cs_{0.58}Co[Fe]_{0.85}$	0.49	135.9	127.6	6.059	1.0178	S23
$Cs_{0.98}Co[Fe]_{0.98}$	0.96	134.8	126.1	6.411	1.0206	S23
$Rb_{0.45}Co[Fe]_{0.82}$	0.37	134.3	125.0	6.922	0.9776	S24

Table S1: Samples used to generate Figs. 1(c) and 2(a) in the main text. Molecular formula is simplified from $A_xM[M'(CN)_6]_y{\cdot}zH_2O$ to $A_xM[M']_y$

2 Discussion regarding E_{Coul}

In the main text, we claim that distortions of the BX_3 framework that act to reduce the A–X distance (by d, say) give rise to electrostatic energy changes that are approximately quadratic in d. By virtue of the symmetry of the X site in the aristotype structure, the crystal energy must be invariant with respect to the substitution $d \rightarrow -d$ and hence any expansion of the energy in terms of d can contain even powers only. In this respect, the quadratic term is the leading term in the energy expansion and must dominate at low d.

As a numerical illustration that the quadratic term likely dominates over all sensible displacements d, we used the GULP code[S25] to calculate the lattice energy for a model two-dimensional perovskite ABX_2 as a function of displacement d. This model represents the schematic drawn in Fig. 1(a) of the main text. The corresponding GULP input was

```
prop conv
title
abx2
end
cell
7.0710678 7.0710678 10.0  90.000000  90.000000 90.0
frac
A  0.0 0.5 0.0 1.0
B  0.0 0.0 0.0 1.0
X  0.75+d 0.25+d 0.0 -1.0
space
140
```

Note that the only interactions included in the lattice energy calculation are electrostatic in origin. A plot of the lattice energy as a function of d is shown in Fig. S1, from which it is clear that the electrostatic contribution varies with d^2 as claimed in the main text. In these calculations, $-0.05 \leq d \leq 0.05$, which corresponds to displacements of about 0.4 Å.

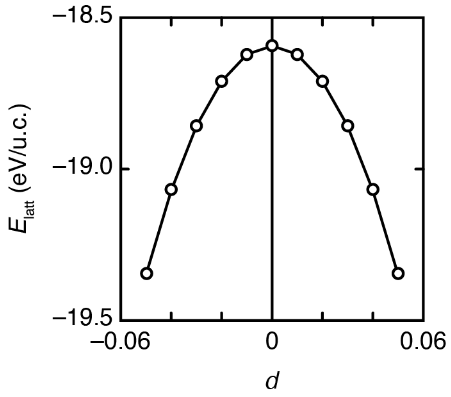

Figure S1: Variation in lattice energy with displacement magnitude for a simple two-dimensional perovskite analogue, as calculated using GULP.

3 References

(S1) Cattermull, J.; Pasta, M.; Goodwin, A. L. Structural Complexity in Prussian Blue Analogues. *Mater. Horiz.* **2021**, *8* 3178–3186.

(S2) Cattermull, J.; Roth, N.; Cassidy, S. J.; Pasta, M.; Goodwin, A. L. K-ion slides in Prussian Blue Analogues. *ChemRxiv* **2023**; doi:10.26434/chemrxiv-2023-gs41m.

(S3) Her, J. H.; Stephens, P. W.; Kareis, C. M.; Moore, J. G.; Min, K. S.; Park, J. W.; Bali, G.; Kennon, B. S.; Miller, J. S. Anomalous Non-Prussian Blue Structures and Magnetic Ordering of $K_2Mn^{II}[Mn^{II}(CN)_6]$ and $Rb_2Mn^{II}[Mn^{II}(CN)_6]$. *Inorg. Chem.* **2010**, *49*, 1524–1534.

(S4) Cattermull, J.; Sada, K.; Hurlbutt, K.; Cassidy, S. J.; Pasta, M.; Goodwin, A. L. Uncovering the Interplay of Competing Distortions in the Prussian Blue Analogue $K_2Cu[Fe(CN)_6]$. *Chem. Mater.* **2022**, *34*, 5000–5008.

(S5) Hosaka, T.; Fukabori, T.; Kojima, H.; Kubota, K.; Komaba, S. Effect of Particle Size and Anion Vacancy on Electrochemical Potassium Ion Insertion into Potassium Manganese Hexacyanoferrates. *ChemSusChem* **2021**, *14* 1166–1175.

(S6) Jiang, L.; Lu, Y.; Zhao, C.; Liu, L.; Zhang, J.; Zhang, Q.; Shen, X.; Zhao, J.; Yu, X.; Li, H.; Huang, X.; Chen, L.; Hu, Y. S. Building Aqueous K-Ion Batteries for Energy Storage. *Nat. Energy* **2019**, *4*, 495–503.

(S7) Deng, L.; Qu, J.; Niu, X.; Liu, J.; Zhang, J.; Hong, Y.; Feng, M.; Wang, J.; Hu, M.; Zeng, L.; Zhang, Q.; Guo, L.; Zhu, Y. Defect-Free Potassium Manganese Hexacyanoferrate Cathode Material for High-Performance Potassium-Ion Batteries. *Nat. Commun.* **2021**, *12*, 2167.

(S8) Bie, X.; Kubota, K.; Hosaka, T.; Chihara, K.; Komaba, S. A Novel K-Ion Battery: Hexacyanoferrate(II)/Graphite Cell. *J. Mater. Chem. A* **2017**, *5*, 4325–4330.

(S9) Li, C.; Wang, X.; Deng, W.; Liu, C.; Chen, J.; Li, R.; Xue, M. Size Engineering and Crystallinity Control Enable High-Capacity Aqueous Potassium-Ion Storage of Prussian White Analogues. *ChemElectroChem* **2018**, *5*, 3887–3892.

(S10) He, G.; Nazar, L. F. Crystallite Size Control of Prussian White Analogues for Nonaqueous Potassium-Ion Batteries. *ACS Energy Lett.* **2017**, *2*, 1122–1127.

(S11) Jiang, X.; Zhang, T.; Yang, L.; Li, G.; Lee, J. Y. A Fe/Mn-Based Prussian Blue Analogue as a K-Rich Cathode Material for Potassium-Ion Batteries. *ChemElectroChem* **2017**, *4*, 2237–2242.

(S12) Xue, L.; Li, Y.; Gao, H.; Zhou, W.; Lü, X.; Kaveevivitchai, W.; Manthiram, A.; Goodenough, J. B. Low-Cost High-Energy Potassium Cathode. *J. Am. Chem. Soc.* **2017**, *139*, 2164–2167.

(S13) Boström, H. L. B.; Collings, I. E.; Daisenberger, D.; Ridley, C. J.; Funnell, N. P.; Cairns, A. B. Probing the Influence of Defects, Hydration, and Composition on Prussian Blue Analogues with Pressure. *J. Am. Chem. Soc.* **2021**, *143*, 3544–3554.

(S14) Matsuda, T.; Kim, J.; Moritomo, Y. Control of the Alkali Cation Alignment in Prussian Blue Framework. *Dalt. Trans.* **2012**, *41*, 7620–7623.

(S15) Ohkoshi, S. I.; Tokoro, H.; Hashimoto, K. Temperature- and Photo-Induced Phase Transition in Rubidium Manganese Hexacyanoferrate. *Coord. Chem. Rev.* **2005**, *249* (1830-1840).

(S16) Boström, H. L. B.; Smith, R. I. Structure and Thermal Expansion of the Distorted Prussian Blue Analogue $RbCuCo(CN)_6$. *Chem. Commun.* **2019**, *55*, 10230–10233.

(S17) Chapman, K. W.; Chupas, P. J.; Kepert, C. J. Compositional Dependence of Negative Thermal Expansion in the Prussian Blue Analogues $M^{II}Pt^{IV}(CN)_6$ (M = Mn, Fe, Co, Ni, Cu, Zn, Cd). *J. Am. Chem. Soc.* **2006**, *128*, 7009–7014.

(S18) Buser, J. H.; Schwarzenbach, D.; Petter, W.; Ludi, A. The Crystal Structure of Prussian Blue: $Fe_4[Fe(CN)_6]_3{\cdot}xH_20$. *Inorg. Chem.* **1977**, *16*, 2704–2710.

(S19) Boström, H. L. B.; Collings, I. E.; Cairns, A. B.; Romao, C. P.; Goodwin, A. L. High-Pressure Behaviour of Prussian Blue Analogues: Interplay of Hydration, Jahn-Teller Distortions and Vacancies. *Dalt. Trans.* **2019**, *48*, 1647–1655.

(S20) Cattermull, J.; Wheeler, S.; Hurlbutt, K.; Pasta, M.; Goodwin, A. L. Filling Vacancies in a Prussian Blue Analogue Using Mechanochemical Post-Synthetic Modification. *Chem. Commun.* **2020**, *56*, 7873–7876.

(S21) Ojwang, D. O.; Grins, J.; Wardecki, D.; Valvo, M.; Renman, V.; Häggström, L.; Ericsson, T.; Gustafsson, T.; Mahmoud, A.; Hermann, R. P.; Svensson, G. Structure Characterization and Properties of K-Containing Copper Hexacyanoferrate. *Inorg. Chem.* **2016**, *55*, 5924–5934.

(S22) Moritomo, Y.; Kato, K.; Kuriki, A.; Takata, M.; Sakata, M.; Tokoro, H.; Ohkoshi, S. I.; Hashimoto, K. Structural Transition Induced by Charge-Transfer in $RbMn[Fe(CN)_6]$ - Investigation by Synchrotron-Radiation X-Ray Powder Analysis. *J. Phys. Soc. Jpn.* **2002**, *71*, 2078–2081.

(S23) Escax, V.; Bleuzen, A.; Cartier dit Moulin, C.; Villain, F.; Goujon, A.; Varret, F.; Verdaguer, M. Photoinduced Ferrimagnetic Systems in Prussian Blue Analogues $C^I_xCo_4[Fe(CN)_6]_y$ (C^I = Alkali Cation). 3. Control of the Photo- and Thermally Induced Electron Transfer by the $[Fe(CN)_6]$ Vacancies in Cesium Derivatives. *J. Am. Chem. Soc.* **2001**, *123*, 12536–12543.

(S24) Bleuzen, A.; Lomenech, C.; Escax, V.; Villain, F.; Varret, F.; Cartier Dit Moulin, C.; Verdaguer, M.

Photoinduced Ferrimagnetic Systems in Prussian Blue Analogues $C^{I}_{x}Co_4[Fe(CN)_6]_y$ (C^{I} = Alkali Cation). 1. Conditions to Observe the Phenomenon. *J. Am. Chem. Soc.* **2000**, *122*, 6648–6652.

(S25) Gale, J. D.; Rohl, A. L. The General Utility Lattice Program (GULP). *Mol. Simul.* **2003**, *29*, 291–341.
